# A case report of vanishing bile duct syndrome after exposure to pexidartinib (PLX3397) and paclitaxel

**DOI:** 10.1038/s41523-019-0112-z

**Published:** 2019-06-14

**Authors:** Sorbarikor Piawah, Colby Hyland, Sarah E. Umetsu, Laura J. Esserman, Hope S. Rugo, A. Jo Chien

**Affiliations:** 1 Department of Medicine, Helen Diller Family Comprehensive Cancer Center, University of California San Francisco, San Francisco, CA USA; 2 Department of Surgery, Helen Diller Family Comprehensive Cancer Center, University of California San Francisco, San Francisco, CA USA; 3 Department of Pathology, University of California San Francisco, San Francisco, CA USA

**Keywords:** Breast cancer, Drug development

## Abstract

Pexidartinib (PLX3397) is a small molecule tyrosine kinase and colony-stimulating factor-1 inhibitor with FDA breakthrough therapy designation for tenosynovial giant-cell tumor, and currently under study in several other tumor types, including breast cancer, non-Hodgkin’s lymphoma, and glioblastoma. Here, we report a case of severe drug-induced liver injury requiring liver transplantation due to vanishing bile duct syndrome (VBDS) after exposure to pexidartinib in the I-SPY 2 Trial, a phase 2 multicenter randomized neoadjuvant chemotherapy trial in patients with Stage II–III breast cancer. We also review the current literature on this rare, idiosyncratic, and potentially life-threatening entity.

## Introduction

Drug-induced liver injury (DILI) is a growing medical and public health problem. With a worldwide estimated annual incidence of 1–2 severe cases per 1000–10,000 patients exposed, it is the most common cause of acute liver failure in the United States.^1,2^ Because most new drugs are tested in fewer than 3000 people by the time a new drug application is filed, cases of severe DILI are usually discovered only after drug approval.

Here, we report a case of severe DILI requiring liver transplantation after exposure to pexidartinib (PLX3397), an oral agent used in one experimental arm of the I-SPY 2 platform trial of neoadjuvant study for early breast cancer at high risk of early recurrence (clinicaltrials.gov identifier NCT01042379). Pexidartinib is a small molecule inhibitor of the c-kit tyrosine kinase and colony-stimulating factor-1 (CSF-1) receptor kinase.^3,4^ CSF-1 has been linked to tumor growth and progression in breast cancer,^5,6^ and has been shown to effectively reduce the number of tumor-associated macrophages in different tumor types.^4,5^

Pexidartinib was granted Breakthrough Therapy Designation by the U.S. Food and Drug Administration (FDA) in 2015 for tenosynovial giant-cell tumor (TGCT) based on phase 1 results that showed significant response;^4^ it is currently under FDA review for the treatment of TGCT based on a phase 3 registration study.^7^ Pexidartinib is also being evaluated in several other cancer types, including refractory Hodgkin’s lymphoma (NCT01217229), glioblastoma multiforme (NCT01349036), and metastatic breast cancer (NCT01596751). Studies have reported elevated aminotransferases in ~50% of patients, generally considered to be a consequence of CSF-1 pathway inhibition in Kupffer cells in the liver, and the most common adverse effect of pexidartinib.^4,8^ Other reported toxicities include hair color changes, periorbital edema, vomiting, fatigue, and dysgeusia.^7^

## Results

### Patient history and presentation

The patient is a 62-year-old postmenopausal woman diagnosed with a clinical stage 2B hormone receptor positive, HER2-negative left breast cancer. Primary tumor was 3 cm with a 2.5–cm mobile left axillary lymph node. She was otherwise healthy with no significant past medical history. Reported medications included vitamin D-3, citalopram, and zolpidem, and the patient denied use of over-the-counter medications, including herbs and supplements. On average, she reported consumption of seven glasses of wine/week. Her BMI was 30. Baseline liver function tests (LFTs) were in the normal range. Staging PET/CT showed no evidence of distant metastasis.

The patient provided written informed consent prior to screening and again prior to enrollment in the I-SPY 2 TRIAL,^9–11^ a trial testing agents in combination chemotherapy in the neoadjuvant setting in patients with early breast cancer. The trial was approved by the UCSF Institutional Review Board, and written informed consent was obtained for publication of this case report. The goal of neoadjuvant chemotherapy in the treatment of early-stage breast cancer is to down-stage tumors, improve surgical options, and to assess response to therapy which provides valuable prognostic information. Patients in this multi-arm trial are randomized to receive standard chemotherapy or standard chemotherapy in combination with one of several experimental agents. The patient was randomized to an investigational treatment arm including weekly intravenous paclitaxel (80 mg/m^2^) in combination with oral pexidartinib 1200 mg daily for 12 weeks. The dose of pexidartinib was selected based on safety and efficacy data from a previously reported phase 1b trial of pexidartinib and weekly paclitaxel in patients with advanced solid tumors.^12^

Approximately 3 weeks after starting study therapy (3 doses of weekly paclitaxel and 27 oral daily doses of pexidartinib), the patient was admitted for fever up to 103 °F. Initial laboratory studies were notable only for mildly elevated transaminases (ALT 65 U/L, AST 105 U/L) with normal white blood cell count, alkaline phosphatase (ALP), and total bilirubin (Tbili). The patient discontinued all study therapy immediately.

### Diagnostic workup

Infectious workup, including blood and urine cultures, viral panel, and chest X-ray were unremarkable. The patient remained febrile to 103.5 °F. LFTs continued to rise during the hospitalization, with day 3 studies showing ALT 158 U/L, AST 188 U/L, ALP 119 U/L, and Tbili 1.9 mg/dL (direct bilirubin 1.3 mg/dL). On hospital day (HD) 3, an abdominal ultrasound showed evidence of gallbladder wall thickening and trace peri-cholecystic fluid, but no evidence of cholelithiasis or biliary ductal dilatation. Abdominal/pelvis CT scan showed new diffuse heterogeneous enhancement of the liver with periportal edema and marked gallbladder wall thickening.

Following the abdominal/pelvis CT scan, the patient was started on broad spectrum antibiotics for empiric treatment of acalculous cholecystitis. Hepatobiliary (HIDA) scan showed no focal perfusion defects and was consistent with cholestasis and a high functional obstruction of the common and cystic duct. Magnetic resonance cholangiopancreatography (MRCP) revealed heterogeneous enhancement of the liver without focal liver lesions. There was no intra- or extrahepatic bile duct dilatation and no contrast excretion into the biliary system.

The patient underwent laparoscopic cholecystectomy on HD 6. An intraoperative cholangiogram was performed and did not show significant filling defects, extravasation, or dilatation of the visualized intra- and extrahepatic ducts. The final surgical pathology was consistent with acute cholecystitis.

The LFTs continued to increase post cholecystectomy, with a rise in bilirubin out of proportion to the rise in transaminases. On HD10, ALT was 194 U/L, AST 250 U/L, ALP 377 U/L, and Tbili 10.9 mg/dL. Due to the continued steady rise in bilirubin, a diagnostic endoscopic retrograde cholangiopancreatography (ERCP) was performed on HD10, which revealed a cannulated common bile duct and normal appearing bile duct on cholangiogram. The intrahepatic biliary tree was filled and appeared normal.

**Fig. 1:**
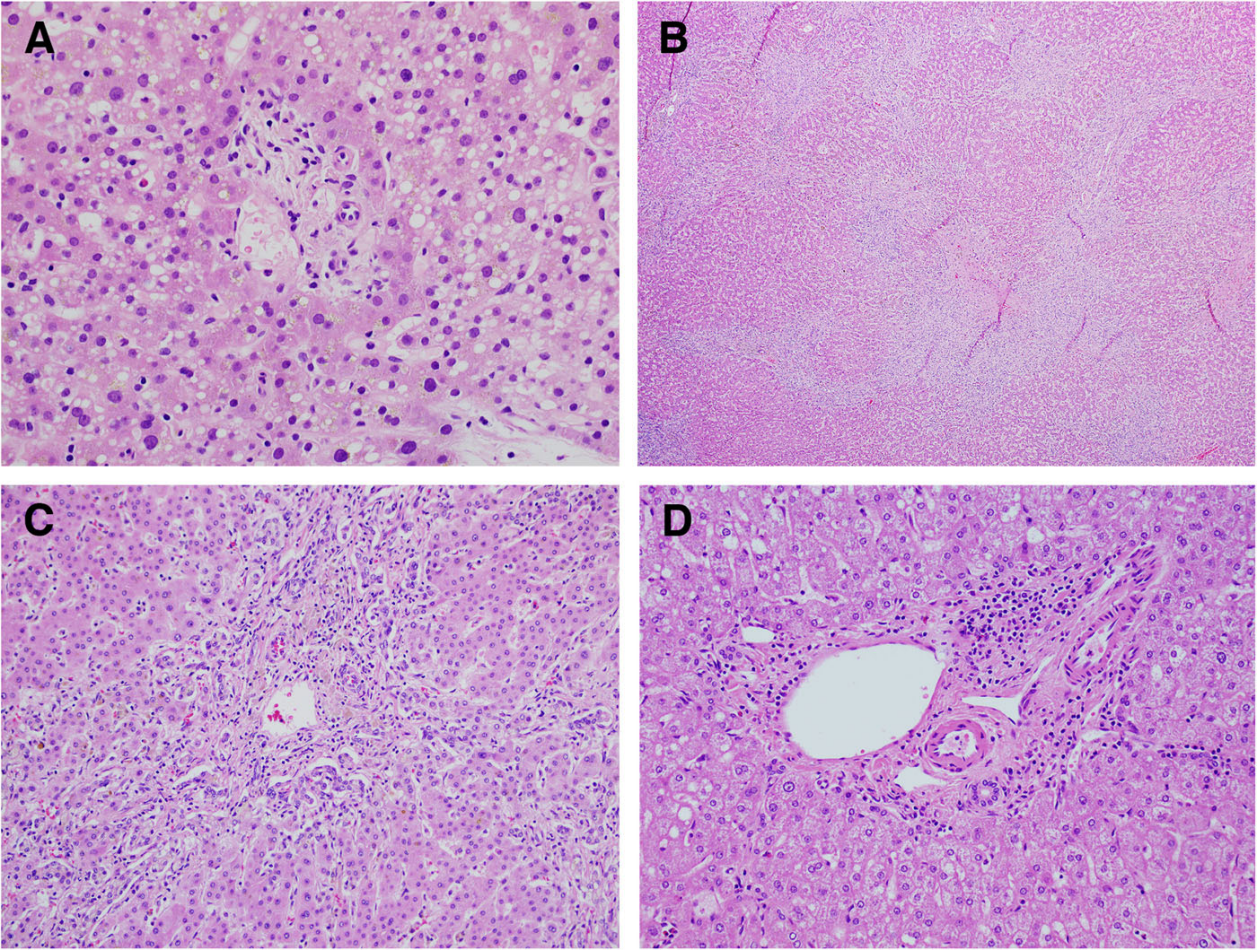
Histology. **a** Representative portal tract from the liver biopsy with absence of the interlobular bile duct. The hepatocytes show severe small droplet steatosis. (H&E, ×400). **b** The explant liver showed a biliary pattern of cirrhosis with extensive ductular reaction (H&E, ×40). **c** A portal tract from the liver explant with absence of the interlobular bile duct and associated ductular reaction and cholestasis (H&E, ×20). **d** Example of normal portal tract

A liver core biopsy was performed on HD 11. The biopsy revealed mild cholestasis with small and attenuated bile ducts and focal duct loss. Representative images are shown in Fig. 1, along with a normal liver biopsy for comparison. The portal tracts contained minimal lymphocytic inflammation and no ductular reaction. The lobules showed grade 3 small droplet steatosis without ballooned hepatocytes or Mallory hyaline, and no significant hepatocyte necrosis. In particular, there was no evidence of malignancy. Trichrome stain showed no increased fibrosis. Copper stain was negative, and iron showed 1 + staining in hepatocytes. Overall the findings were of cholestasis with duct damage, and duct loss (only three of eight portal tracts containing ducts) (Fig. 1a). Based on these findings and the patient’s clinical presentation, a diagnosis of acute drug-induced liver injury, suggestive of vanishing bile duct syndrome (VBDS) was made, most likely secondary to the experimental agent pexidartinib in combination with paclitaxel.

### Medical management and treatment

The patient was started on ursodiol 1200 mg/day for progressive pruritis on HD 12. Prednisone 40 mg daily was started on HD 14, and she was discharged to home on HD 15.

Following discharge, the patient was tapered off prednisone over the course of 1 month. She was started on the aromatase inhibitor, letrozole, 2 weeks after discharge from the hospital. Despite initial improvement in transaminases and ALP, her Tbili continued to rise and remained persistently elevated to a peak of 32.6, 5 months after discontinuation of pexidartinib. Six months after starting letrozole, she underwent left mastectomy and axillary lymph node dissection. Pathology revealed residual 2 -cm grade 2 invasive ductal carcinoma with a cellularity of 5%. Notably, 20 axillary nodes were negative for carcinoma demonstrating an excellent response to neoadjuvant systemic therapy.

Over the following 13 months, the patient’s LFTs remained abnormal with ALT/AST 150–200 U/L, ALP 350–400 U/L, and Tbili 20–25 mg/dL. During this time, her functional status deteriorated due to progressive fatigue, anorexia, pruritis, and depression. Greater than 50% of her time was spent in bed, and she lost 60 pounds. She remained on ursodiol and doxepin for pruritis, as well as monthly vitamin K injections. Due to chronicity and severity of her symptoms, the patient underwent orthotopic liver transplant 20 months after her initial diagnosis of severe DILI.

The liver explant showed cirrhosis with cholestasis, loss of interlobular bile ducts, and extensive ductular reaction. Larger bile ducts appeared intact without duct damage. No significant steatosis was seen (grade 0). Overall, the findings were consistent with ductopenia, likely secondary to drug-induced vanishing bile duct syndrome (Fig. 1b, c).

**Fig. 2:**
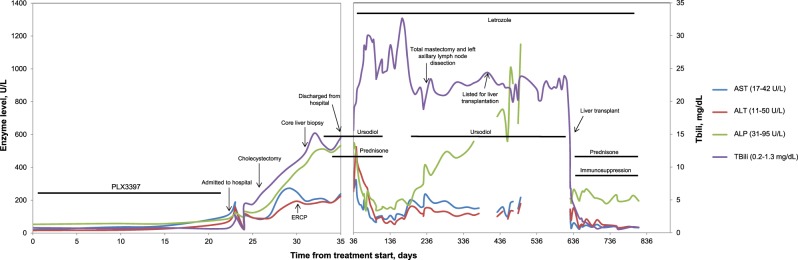
Trends in hepatic enzyme and bilirubin levels in U/L from baseline through 6 months post-liver transplant. Aspartate aminotransferase (AST), alanine transaminase (ALT), and alkaline phosphatase (ALP) trends are shown on the primary *y* axis on the left. The total bilirubin (TBili) trends are shown on the secondary *y* axis on the right

Following transplantation, the patient’s LFTs and performance status improved precipitously. She is currently on chronic immunosuppression with everolimus and is thriving clinically 22 months post transplant. Her appetite and energy have significantly improved, pruritis has resolved, and she is at a healthy weight. She remains disease free from breast cancer on adjuvant letrozole. The trend in her LFTs pre- and post transplant are outlined in Fig. 2.

## Discussion

VBDS is a form of DILI that describes acquired disorders related to the progressive destruction and disappearance of intrahepatic bile ducts, leading to cholestasis.^13^ First described in 1988 in three patients,^14^ VBDS is largely idiosyncratic, but has been causally associated with a variety of etiologies, including genetic diseases such as cystic fibrosis and Williams syndrome,^15^ neoplasms such as lymphoma,^16–18^ immune disorders including graft-versus-host disease,^15,19^ viral infections,^20–23^ and most commonly, pharmaceuticals. VBDS has been associated with numerous drug classes, including antibiotics, non-steroidal anti-inflammatories, antipsychotics, antiepileptics, antihypertensives, diabetes medications,^13,24–33^ and oral chemotherapy drugs such as temozolomide.^30,31^

In most instances, the ductopenia in VBDS preferentially affects the smaller interlobular bile ducts rather than the larger bile ducts that are usually destroyed in primary biliary cirrhosis. Defined as the loss of >50% of interlobular ducts, or a bile duct to portal tract ratio of <0.5,^2,14,33–35^ patients with VBDS commonly present with jaundice, itching, fatigue, and anorexia.^2,31,32^ Laboratory evaluation reveals cholestasis, including profound elevations in ALP and gamma-glutamyl transferase, as well as high serum concentrations of bilirubin, with relatively mild elevations in serum aminotransferases.^2^ It is hypothesized that the bile duct injury seen in VBDS is due to an inflammatory response directed at cholangiocytes, which play an integral role in lining the lumen of bile ducts, sealing the biliary epithelium, and bile formation. Immune-mediated destruction of cholangiocytes results in prolonged cholestasis, bile duct degeneration, and loss of bile ducts.^2^ In the acute phase, histologically there may be evidence of periportal mixed inflammatory infiltrates including neutrophils, lymphocytes, and eosinophils, as well as cytoplasmic vacuolization and swelling of cholangiocytes and ductular infiltration by lymphocytes.^2,35^ As this inflammatory response diminishes following discontinuation of the offending agent, in most cases cholestasis improves. However, in a minority of patients, such as ours, progressive bile duct loss becomes chronic and results in liver transplant, or death.^17,32,35^

There are limited data regarding the optimal treatment for VBDS. In general, treatment is supportive, focused on reducing symptoms associated with prolonged cholestasis. Ursodeoxycholic acid is a mainstay of therapy, and is thought to stimulate bile secretion and promote survival of cholangiocytes by inhibiting the intrinsic apoptosis pathway.^2,36^ Steroids and other immunosuppressants such as low-dose mycophenolate have been used successfully, suggesting a potential benefit in targeting the underlying inflammatory mechanisms described above.^2,37^

There are limited data regarding the prognosis of VBDS. Some insight is provided by the Drug Induced Liver Injury Network (DILIN) study. In this prospective study of 363 patients with a diagnosis of DILI and a liver biopsy, 26 patients (7%) had evidence of bile duct loss, consistent with VBDS.^32^ The time from starting the implicated drug to onset of VBDS symptoms ranged from 3-551 days. Patients with evidence of bile duct loss on biopsy were more likely to develop cholestatic chronic liver injury (94 vs. 47%) and had significantly higher mortality rates (27 vs. 9%) compared with the 337 patients with DILI without evidence of VBDS.^32^ The most significant predictor of a poor outcome, including death or liver transplantation, was the degree of bile duct loss in the diagnostic liver biopsy. Patients with poor outcomes had a lower average percent of portal areas with bile ducts present compared with patients with good outcomes (17 vs. 64%, respectively). Laboratory tests at the time of injury were not predictive of outcome.^32^

Here, we report VBDS and severe DILI associated with pexidartinib, in this case, combined with weekly paclitaxel. Given the extensive existing safety data on paclitaxel (with cholestatic jaundice infrequently reported),^38^ it is unlikely that paclitaxel was the cause of this patient’s VBDS and severe DILI; however, it is possible that paclitaxel played a role in combination with pexidartinib. This incident is a reminder that close monitoring of LFTs for patients enrolled on clinical trials is of critical importance, as prior experience may not uncover these rare events. Some cases of severe drug-induced DILI cannot be halted or reversed, despite vigilance and early discontinuation of the offending agent—it is worth noting that in this report, pexidartinib was discontinued at the earliest sign of toxicity, yet cholestasis was irreversible. As we continue to evaluate new targeted agents in patients with potentially curable malignancies, it is critical that safety be monitored in real time with immediate feedback to reduce potentially life-threatening toxicities. Further evaluation of these rare, idiosyncratic cases of severe DILI/VBDS will help identify patients that may be at particular risk, and help improve the prognosis for affected patients.

### Reporting summary

Further information on research design is available in the Nature Research Reporting Summary linked to this article.

## Supplementary information

Reporting Summary

## Data Availability

Datasets that support the figures presented in this paper are available upon request, as described at: 10.6084/m9.figshare.8089343.^39^

## Supplementary information

**Supplementary Information** accompanies the paper on the *npj Breast Cancer* website (10.1038/s41523-019-0112-z).
